# Body Weight Gain Status during the Incubator Weaning Process in Very Low Birth Weight Premature Infants

**DOI:** 10.3390/children9070985

**Published:** 2022-06-30

**Authors:** Chung-Wei Lin, Hsiang-Yun Ko, Chih-Chi Huang, Chiu-Yu Yeh, Yen-Chun Chiu, Hsiu-Lin Chen

**Affiliations:** 1 School of Medicine, College of Medicine, Kaohsiung Medical University, Kaohsiung 807, Taiwan; russellcwlin@gmail.com (C.-W.L.); kohsiangyun@gmail.com (H.-Y.K.); u105001111@gap.kmu.edu.tw (C.-C.H.); carolyn0503@gmail.com (C.-Y.Y.); sean.sep17@hotmail.com (Y.-C.C.); 2 Department of Pediatrics, Kaohsiung Medical University Hospital, Kaohsiung 807, Taiwan; 3 Department of Respiratory Therapy, College of Medicine, Kaohsiung Medical University, Kaohsiung 807, Taiwan

**Keywords:** calories status, incubator, newborn, premature infant, very low birth weight, weaning

## Abstract

Incubator care is essential for premature infants during early hospitalization. As the infants’ conditions improve, incubator weaning becomes necessary. This retrospective study aimed to evaluate the effect of body weight gain and status of intake-calorie gain on the incubator weaning process for very low birth weight (VLBW) premature infants. The study included 127 VLBW premature neonates. We analyzed data on clinical characteristics potentially associated with the weaning period and the end-weaning body weight (EWBW), including body weight gain status, intake-calorie gain status, and disease conditions. The neonates were weaned from the incubators at a mean postmenstrual age (PMA) of 35.1 ± 1.3 weeks; postnatal days, 37.7 ± 18.2 days; and body weight, 1882.8 ± 157.1 g. The total weaning period was 3.5 ± 3.1 days. Regarding the weaning period, there was a strong positive relationship only in the end-weaning PMA and the daily body weight within 3 days before incubator weaning. Further, regarding the factors associated with EWBW, only the end-weaning PMA and necrotizing enterocolitis had a significant positive impact. Body weight gain and the status of intake-calorie gain showed no association with either the weaning period or the EWBW and, thus, were not related to the incubator weaning process.

## 1. Introduction

Premature newborns are born alive before the completion of 37 weeks of pregnancy. Due to their premature development, they are often in need of more intensive care in incubators, which helps them grow in the early period after birth. Given that the global preterm birth rate has been rising in the past 20 years [1,2], great progress in incubator care has also benefited the developmental needs of neonates with respect to many clinical aspects. Among the benefits that the incubators provide, thermal maintenance is one of the most crucial supports for the premature infants to maintain basic physiological functions. In conjunction with incubator care, other interventions, such as feeding support, infection prevention, safe oxygen use, good quality neonatal resuscitation, and kangaroo mother care are equally important for close monitoring of preterm infants [3]. These interventions ensure better early development in preterm neonates.

As the premature infants reach a relatively stable status in the intensive care unit, the physicians usually try weaning the infants from the incubators. Customarily, after the “try weaning” decision is made, certain observational tests are essential to evaluate the physiological condition of the infant. This prevents abrupt incubator weaning decisions that may lead to the worsening of outcomes. For instance, the clothing test [4], a common maneuver, reflects the early adaptability of premature newborns to the environmental temperature. By keeping the body temperature relatively stable to detect if there are abnormal physiological changes, the clothing test serves as a meaningful indicator for safe and successful weaning of the neonate from the incubator. In addition, the clothing tests during the weaning period give the physicians clear and detailed insights into the clinical situation of the premature newborns on a daily basis. This weaning process does not only help physicians prepare for the upcoming incubator weaning of the infants, but also helps them distribute resources among individuals more effectively.

Currently, in clinical practice, most premature newborns are considered candidates for clothing tests if they are clinically stable. However, clear criteria regarding the body weight threshold for clothing tests and the final incubator weaning have not yet been established; instead, the timing and length of the weaning period still primarily depend on the clinical experiences and professional judgement of the medical staff [5]. In addition, it is unclear how the key elements of the infants’ growth and the body weight gain and intake-calorie gain status influence the weaning decision during the clothing tests. Therefore, this study aimed to investigate the correlations between clinical features and weaning outcomes and the body weight at the end of clothing tests (end-weaning body weight, EWBW) in premature newborns with successful weaning experiences. The study sought to provide a better understanding of the mechanisms associated with incubator weaning issues and aid the preparation for incubator weaning in the future.

## 2. Materials and Methods

### 2.1. Study Design and Data Collection

This retrospective study was conducted in accordance with the Declaration of Helsinki, and the protocol was approved by the Institutional Review Board (IRB) of Kaohsiung Medical University Hospital (IRB No. KMUHIRB-SV(I)-20210042, Date 18 June 2021). We did not obtain informed consent for this study because this retrospective study used medical chart data without contacting the parents. Since there were limited previous articles discussing the issue we investigated, neither a formal nor a similar model could be adopted for sample size calculation; hence, we assumed that at least a hundred participants would constitute a representative population. A total of 127 preterm neonates with birth body weights below 1500 g were included from the neonatal intensive care unit (NICU) of Kaohsiung Medical University Hospital during the period of 2017–2019, after excluding death cases and those with congenital anomalies. All the neonates were nursed in incubators from birth and weaning from the incubators was attempted later as scheduled. All the preterm neonates reported in this study eventually weaned successfully from the incubators.

**Figure 1:**
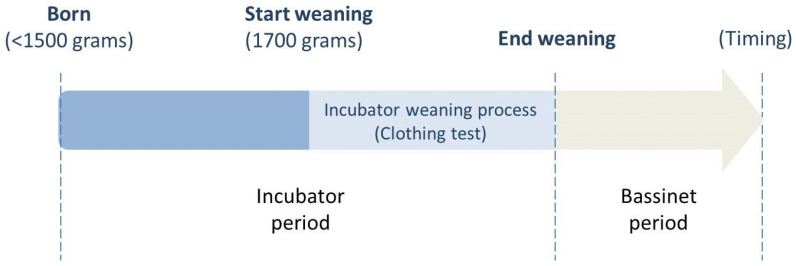
Clinical practice of care for preterm neonates. The timeline shows the progress of care management in preterm neonates. The preterm neonates born under 1500 g are initially cared for in incubators. After their body weight reaches approximately 1700 g without serious active medical problems (e.g., apnea), a clothing test will be administered in the incubators. Eventually, at the end of the clothing tests, they are weaned from the incubators to the bassinets if the body temperature can be maintained at approximately 36.5–37 °C and no complications occur.

Data on basic and clinical characteristics of the 127 preterm neonates were collected. Basic characteristics included sex, gestational age (GA), delivery methods, multiple births, Apgar scores one and five minutes after birth, birth body weight (BBW), and small for gestational age (SGA). Clinical characteristics included diseases, such as respiratory distress syndrome (RDS), patent ductus arteriosus (PDA), necrotizing enterocolitis (NEC), bronchopulmonary dysplasia (BPD), and retinopathy of prematurity (ROP), postmenstrual age (PMA), postnatal days (PND), and body weight at the beginning (start weaning) and end (end of weaning) of clothing tests. In addition, we collected data on the total weaning period (in days), the z-score of Fenton growth weight at incubator weaning, as well as the status for intake-calorie and body weight within 3 days before incubator weaning. The description of intake-calorie/body weight (gain) status is as follows: daily intake-calorie/body weight indicates the mean of intake-daily calories/body weight within 3 days before incubator weaning; daily change of intake-calorie/body weight indicates the mean difference of intake-calorie/body weight within 3 days before incubator weaning; and the variation of daily change of intake-calories/body weight indicates the variation of the mean difference of intake-calorie/body weight within 3 days before incubator weaning. The body weight at the beginning of clothing tests (start weaning) was presented as the start-weaning body weight (SWBW), and the body weight at the end of clothing tests (end of weaning) was presented as the end-weaning body weight (EWBW). The total weaning period was defined as the duration from the beginning of the clothing tests in the double-walled incubator (start weaning) to the day of weaning from the incubator to the bassinet (end of weaning). The primary outcome of our study was whether intake-calorie gain and body weight gain statuses were related to the weaning period (days of clothing test, shown on Figure 1) and EWBW. The secondary outcome was whether there were other clinical factors related to the weaning period and EWBW.

### 2.2. Care System

Regarding the settings of the incubator environment, the thermal control of the incubators was automatically regulated by monitoring the preterm neonate’s skin surface via Servo control to keep it at 36.5 °C, and the relative humidity was maintained at 50–80% as is standard protocol. Fortified human milk was the neonate’s main source of nutrition, with a substitutional commercial formula for supplementing calories as needed. Parenteral nutrition would be administered if any unstable vital signs happened to alter the digestive function. The calories of different nutritional components were calculated and summed up for daily evaluation and adjustment. Regarding the general rules of intake-calorie administration, nutrition of 50–60 kcal/kg/day was for body weight maintenance, and daily intake-calories were gradually increased to 110–140 kcal/kg/day based on the assessment of the neonates’ daily body weight gain and digestive function. All the daily body weight changes and intake-calories were carefully monitored and recorded separately. According to the practice guideline of our institution (see Figure 1), we generally provided care to preterm neonates born under 1500 g in the double-walled incubators. After their body weight reached approximately 1700 g without serious active medical problems (e.g., apnea), a clothing test would be administered in the double-walled incubators to prepare for weaning and for checking the neonates’ adaptability to the outside environment. With the clothing test, the nursing staff helped the infant get dressed in a layer of clothing with a hat, wrapped in a large towel, and covered with a thin quilt in the incubator. Meanwhile, we decreased the air temperature in the incubators gradually to room temperature, which was around 26 °C. If the infants’ temperature could be maintained at 36.5–37 °C in the incubators at 26 °C for one day, they were the candidates to be weaned from the incubators to the bassinets. The infants would be monitored continuously by EKG monitor and pulse oximeter to check the stability of vital signs. The nursing staff would check the body temperature of the infants every 2 h; if the infants’ temperature were below 36.5 °C, the air temperature of the incubators would be increased. Eventually, the neonates were weaned from the incubators at the end of the clothing tests to the bassinets if the body temperature could be maintained at approximately 36.5–37 °C and no complications occurred.

### 2.3. Statistical Analysis

Continuous variables were presented as mean ± standard deviation (SD), and categorical variables were presented as number and percentage (n (%)). Univariate linear regression was used to analyze individual correlations between variables, including GA, BBW, PMA of start weaning, PMA of end weaning, disease pattern, intake-calorie status at incubator weaning, and body weight at incubator weaning and during the weaning period. In addition, univariate linear regression was performed to analyze the individual correlations between the variables mentioned above, except the daily body weight (gram) within 3 days before incubator weaning and EWBW. Of the analyzed data, we used the standardized beta to present the impact of the variables, and a *p* value < 0.05 was considered statistically significant. Statistical analyses were performed using IBM SPSS Statistics for Windows, version 22 (IBM Corp., Armonk, NY, USA).

## 3. Results

A total of 127 neonates in Kaohsiung Medical University Hospital who met the inclusion criteria were recruited for the analysis. The basic characteristics, including sex, GA, delivery methods, multiple birth, APGAR scores one and five minutes after birth, BBW, SGA percentage, and overall congenital anomalies percentage, are presented in Table 1. The study population comprised similar percentages of sex (male = 48.8%; female = 51.2%). The GA was 29.5 ± 2.5 weeks; 53.2% and 46.8% of the neonates were born under and over 30^+0^ weeks, respectively. The BBW was 1188.9 ± 255.3 g; 27.6% and 72.4% of the neonates were born under and over 1000 g, respectively. The APGAR scores one and five minutes after birth were 5.4 ± 1.9 and 7.2 ± 1.8, respectively. The percentages of cesarean and multiple births were 39.4% and 30.7%, respectively. The percentage of SGA was 34.1%. The clinical characteristics of the disease pattern, the variables of the start- and end-weaning, the total weaning period, the Fenton growth weight at incubator weaning, the intake-calorie (gain) status at incubator weaning, and the body weight (gain) status at incubator weaning are presented in Table 2. Of the neonates that weaned successfully from the incubators, the start-weaning PMA was 34.5 ± 1.3 weeks; the start-weaning PND was 35.3 ± 18.1 days; and the SWBW was 1794.7 ± 119.2 g. After evaluation using the clothing tests, the neonates ultimately weaned from the incubators at 1882.8 ± 157.1 g (EWBW); the end-weaning PMA was 35.1 ± 1.3 weeks, and the end-weaning PND was 37.7 ± 18.2 days. The total weaning period was 3.5 ± 3.1 days.

We analyzed the correlations between the clinical parameters and the weaning period using univariate linear regression (Table 3). The findings showed that the end-weaning PMA (standardized β = 0.299; *p* = 0.001) and the daily body weight within 3 days before incubator weaning (standardized β = 0.571; *p* < 0.001) had significant positive correlations with the weaning period. When analyzing the correlations between the clinical characteristics and EWBW using univariate linear regression (Table 4), the end-weaning PMA (standardized β = 0.305; *p* = 0.001) and the presence of NEC (standardized β = 0.179; *p* = 0.046) had a significant positive impact. Since the significant variables were not as many as we expected, we did not perform multivariate linear regression analysis for further assessments. The results shown in both Table 3 and Table 4 demonstrate that the status of intake-calorie gain and body weight gain had no significant relationships with either the weaning period or the EWBW.

## 4. Discussion

To the best of our knowledge, this is the first study to primarily investigate the associations between body weight/intake-calorie gain and the incubator weaning process for preterm infants. We focused on the weaning period and the EWBW in this study, and our analyses showed that in general, the status of body weight gain and intake-calorie gain were not related to either the weaning period or EWBW.

In previous studies, the body weight status rather than the body weight “gain” status was mostly regarded as an indicator for incubator weaning since the body weight status was more intuitive for assessments. As an indicator of maturity, the body weight status has widely been proven to reflect a neonates growth velocity and adaptation to its environments [6]. Further, it also serves as an important indicator of the following factors: successful weaning from the incubator to the crib [4], the length of hospital stays [7,8], and the discharge weight [4,7]. Therefore, most of the relevant research has compared weaning infants of different weight ranges to determine if there were any positive or negative effects of weight on weaning in these vulnerable neonates. For instance, a review by Razak indicated that infants transferred to open cots at a weight lower than 1600 g gained more weight and grew better [9]. The adequacy of transferring infants to open cots at around 1500–2000 g has also been suggested in many studies [4,5,7,8,10,11,12]. All the above evidence implies that weaning preterm neonates from incubators at different weight levels is feasible, based on their stable condition at the decision of weaning and a stage of dynamic equilibrium in changes of their physiological parameters (e.g., daily calorie changes and daily body weight changes). In our study, both SWBW (1794.7 ± 119.2 g) and EWBW (1882.8 ± 157.1 g) were relatively higher compared to the findings of studies from other medical institutions. This implied that the neonates were more qualified for the following clothing tests and weaning, since they were more, or at least as clinically stable, as those being weaned at a lower weight. This concept may explain why the body weight “gain” (change) status, which is an indicator of physiological stability, did not correlate with both the weaning period and the EWBW in our study, since the neonates were equally stable at this stage.

As earlier mentioned, the role of the body weight “gain” status, which reflects the stability of daily weight changes in the incubator weaning process, has been comparatively understudied compared to body weight status. The most current studies principally considered body weight gain as an outcome after successful transfer from incubators [4,6,10,13] with the follow-up period varying among the studies. Zecca et al. [7] compared infants who were weaned from incubators at 1600 g and 1800 g and found that there was no difference in weight gain for over 1 week after transfer. A four-cohort study conducted by West et al. [11] reported that the rate of weight gain was not different before or after the transfer, regardless of the weaning weight. New et al. analyzed the average daily weight gain over the first 14 days following transfer to an open cot, with findings similar to those of previous studies [14]. Though limited, there is also research on the influence of body weight gain on the neonate’s follow-up physiological changes. For example, a clinical trial conducted by Heimler et al. [15] concluded that the weight gain after transfer for over 1 week correlated with the gross energy intake. All the above-mentioned studies demonstrate the importance of body weight gain in development and in the care of preterm neonates, especially at the timing of incubator weaning. Being a continuous process starting from inside the incubator to the external environment, it is quite reasonable to view the changes in body weight outside the incubator as the extension of those occurring within the incubator. Therefore, our research may relate to these previous studies to some extent. Compared to the “start-assessing timing,” (which is defined as the “end-weaning” timing in the current study) and the longer observation durations “after weaning” mentioned in previous studies, we reflected on the weaning process itself and designated “3 days before incubator weaning” and “the end of weaning” as the two respective and precise points in time for assessing the infants’ conditions in our research. This shorter observation duration allowed our medical personnel to record all the data in the hospital, thus avoiding potentially imprecise records obtained from the newborns’ caregiver. This helps us gain more clear insights into the events and mechanisms during the transition period from the incubator to the external environment. Moreover, any abnormal conditions in the health of the neonates could be amplified in a shorter observational period, and thus they could be easily detected and analyzed.

Considering that nutrition is also a critical factor for the development of the infant [16,17] and may directly reflect on the daily weight change [17], the current study also calculated the energy intake amount to evaluate its association with the weaning period and the EWBW. However, no variables regarding the intake-calorie (gain) status discussed in this study were associated to either the weaning period or the EWBW. With regards to a preterm neonate’s basic development, the calorie intake contributes mainly to body weight gain, which leads to the high consistency between these two physiological parameters, i.e., development and weight gain. Hence, it is not surprising that the intake-calorie status was also not related to both the weaning period and the EWBW, which is similar to the previous results of the body weight gain status. Another explanation for this phenomenon is that an energy intake of 120 kcal/kg/day is typically a sign of adequate growth and stable nutritional status for preterm newborns [18,19], and almost all participants in our study met this threshold during the 3 days before weaning with only a small deviation (124.2 ± 8.9 kcal/kg/day). Consequently, it could be expected that the changes in intake-calories would not be significantly manifested under such stable circumstances, which may potentially explain these results that were not statistically significant. However, the changes in calorie intake exert an effect on body weight gain as well as relate to the energy expenditure status, which is difficult to record clinically and should be taken into consideration. For instance, Lei et al. [20] investigated the correlation among body weight, temperature control, and resting energy expenditure (REE) in premature infants on the basis that an increase in the REE may affect optimal body weight gain. This study reported that the REE was significantly elevated in infants during incubator weaning. These results demonstrate that, indeed, the energy output during this period is consequential, and should be monitored for a more comprehensive evaluation. However, we could not assess this parameter in our study due to the lack of technical equipment. Nevertheless, it is important to consider that evaluating the infants’ development and maturity during the weaning process that is solely based on intake-calorie gain may also be relatively imprecise. Aside from body weight and intake-calories, the end-weaning PMA and NEC are two independent factors that were found to be correlated with the weaning process in this study. Because the weaning decision is majorly based on the final body weight status, it is reasonable that the end-weaning PMA was highly correlated with the weaning period and the EWBW. Nonetheless, NEC unexpectedly showed a weak relation in terms of the EWBW (standardized β = 0.179; *p* < 0.046). Being one of the most life-threatening emergencies associated with prematurity, NEC often results in long-term nursing care, and a complicated and costly hospitalization [21]. Hence, it is conceivable that transferring infants with this diagnosis at a heavier body weight is safer. However, no trial relevant to the association of NEC and weaning could be identified and reviewed by our researchers. Moreover, due to the very small sample size of NEC cases (Table 2; 1.6%; two infants only) and the potential effects of the outliers, no additional analysis was performed to further interrogate the exact impact of NEC. Based on the above, we believe that a statistical bias cannot be completely excluded, and future studies with larger sample sizes of NEC cases would better elucidate this topic.

There are some limitations in our study. The first one was the difficulty in estimating a sample size with enough power. Since there are no relevant articles suitable for estimating an adequate sample size, we recruited 127 preterm neonates for analysis. Although significance was demonstrated for NEC in our study, this may be the result of the limited sample size. Thus, future studies with larger samples and adequate statistical power are expected to verify the contributions of the clinical characteristic that we evaluated. Secondly, energy is a critical issue of neonatal development, and is influenced by several factors, including basal metabolism, growth, energy expenditure, and energy losses [22]. Although the REE has been strongly regarded as a potential factor that directly correlates with daily weight gain, it was not measured in our settings due to technical limitations. If, however, we had taken the REE into consideration, we expect that it would have also shared a relationship with the incubator weaning. Lastly, as previously mentioned, we defined “3 days” as a meaningful duration for the bedside observations of the neonates’ body weight status and nutritional status, which is convenient and efficient in clinical practice. However, it is unknown if a longer evaluation period would produce similar or better outcomes, and this needs to be evaluated in future studies.

## 5. Conclusions

Our study demonstrated that the status of body weight gain and intake-calorie gain are not related to the weaning period (days of clothing test) and the EWBW for weaning from the incubator. However, decisions related to the incubator weaning are impacted by the subjective judgment of the clinicians, which are based on the daily body weight status and the clinical situation of premature infants, such as NEC. Therefore, future research is warranted to verify the relationships we identified in this study.

## Figures and Tables

**Table 1:** Basic characteristics at birth for the preterm newborns enrolled in the NICU of Kaohsiung Medical University Hospital.

Basic Characteristics	* Preterm Newborn (*n* = 127)
Sex	
Male	62 (48.8)
Female	65 (51.2)
† GA (week)	29.5 ± 2.5
<30^+0^ weeks	68 (53.2)
≥30^+0^ weeks	59 (46.8)
Delivery (cesarean)	50 (39.4)
Multiple birth	39 (30.7)
APGAR score	
1 min	5.4 ± 1.9
5 min	7.2 ± 1.8
§BBW (gram)	1188.9 ± 255.3
<1000 g	35 (27.6)
≥1000 g	92 (72.4)
‡ SGA	43 (34.1)

* Data are presented either as n (%) or mean ± standard deviation (SD). † GA, gestational age, § BBW, birth body weight, ‡ SGA, small for gestational age, NICU, neonatal intensive care unit.

**Table 2:** Clinical characteristics of preterm newborns enrolled in the NICU of Kaohsiung Medical University Hospital.

Clinical Characteristics	**‡‡** Preterm Newborn (*n* = 127)
**Disease pattern**	
Early sepsis	0 (0)
‡ RDS	123 (96.9)
¶ PDA	40 (30.7)
Ø NEC	2 (1.6)
Ð BPD	16 (12.5)
ð ROP	119 (93.7)
**Start weaning**	
† PMA (week)	34.5 ± 1.3
§ PND (day)	35.3 ± 18.1
Body weight (gram)	1794.7 ± 119.2
**End weaning**	
† PMA (week)	35.1 ± 1.3
§ PND (day)	37.7 ± 18.2
Body weight (gram)	1882.8 ± 157.1
Total weaning period (day)	3.5 ± 3.1
Fenton growth weight at incubator weaning (z-score)	−1.4 ± 0.9
**Intake-calor** **ie status**	
* Daily intake-calories (kcal/kg)	124.2 ± 8.9
**Intake-calorie gain status**	
** Change of daily intake-calories (kcal/kg)	0.9 ± 3.9
*** Variation of change of daily intake-calories (kcal/kg)	5.4 ± 7.2
**Body weight status**	
# Daily body weight (gram)	1816.0 ± 155.8
**Body weight gain status**	
## Change of daily body weight (gram)	37.6 ± 14.5
### Variation of change of daily body weight (gram)	23.3 ± 19.9

‡‡ Data are presented either as n (%) or mean ± standard deviation (SD). ‡ RDS, respiratory distress syndrome; ¶ PDA, patent ductus arteriosus; Ø NEC, necrotizing enterocolitis; Ð BPD, bronchopulmonary dysplasia; ð ROP, retinopathy of prematurity; † PMA, postmenstrual age; § PND, postnatal days; NICU, neonatal intensive care unit; * Mean daily intake-calories within 3 days before incubator weaning; ** Mean difference of intake-calories within 3 days before incubator weaning; *** Variation of mean difference of intake-calories within 3 days before incubator weaning; # Mean daily body weight within 3 days before incubator weaning; ## Mean difference of body weight within 3 days before incubator weaning; ### Variation of mean difference of body weight within 3 days before incubator weaning.

**Table 3:** Correlation of GA, BBW, and clinical characteristics with the weaning periods of the preterm newborns enrolled in the NICU of Kaohsiung Medical University Hospital by means of univariate linear regression.

Preterm Newborn (*n* = 127)	Standardized β	*p* Value
‡‡ **GA (week)**	0.064	0.489
§ BBW (gram)	0.051	0.578
**Disease pattern**		
‡ RDS	0.015	0.873
¶ PDA	0.035	0.706
Ø NEC	0.085	0.351
Ð BPD	−0.014	0.877
ð ROP	−0.050	0.582
† **PMA (week)**		
Start-weaning	0.029	0.753
End-weaning	0.299	0.001
**Intake-calorie status**		
* Daily intake-calories (kcal/kg)	0.049	0.618
**Intake-calorie gain status**		
** Change of daily intake-calories (kcal/kg)	0.141	0.154
*** Variation of change of daily intake-calories (kcal/kg)	−0.010	0.921
**Body weight status**		
# Daily body weight (gram)	0.571	<0.001
**Body weight gain status**		
## Change of daily body weight (gram)	−0.087	0.342
### Variation of change of daily body weight (gram)	0.069	0.455

‡‡ GA = gestational age; § BBW = birth body weight; ‡ RDS =respiratory distress syndrome; ¶ PDA = patent ductus arteriosus; Ø NEC = necrotizing enterocolitis; Ð BPD = bronchopulmonary dysplasia; ð ROP = retinopathy of prematurity; † PMA= postmenstrual age; * Mean daily intake-calories within 3 days before incubator weaning; ** Mean difference of intake-calories within 3 days before incubator weaning; *** Variation of mean difference of intake-calories within 3 days before incubator weaning; # Mean daily body weight within 3 days before incubator weaning; ## Mean difference of body weight within 3 days before incubator weaning; ### Variation of mean difference of body weight within 3 days before incubator weaning.

**Table 4:** Correlation of GA, BBW, and clinical characteristics with the end-weaning body weights of preterm newborns enrolled in the NICU of Kaohsiung Medical University Hospital by means of univariate linear regression.

Preterm Newborn (*n* = 127)	Standardized β	*p* Value
* GA (week)	−0.005	0.956
§ BBW (gram)	0.014	0.876
**Disease pattern**		
‡ RDS	−0.001	0.988
¶ PDA	−0.089	0.325
Ø NEC	0.179	0.046
Ð BPD	−0.065	0.484
Ð ROP	0.024	0.787
† **PMA (week)**		
Start-weaning	0.138	0.134
End-weaning	0.305	0.001
**Intake-calorie status**		
* Daily intake-calories (kcal/kg)	−0.070	0.478
**Intake-calorie gain status**		
** Change of daily intake-calories (kcal/kg)	−0.029	0.771
*** Variation of change of daily intake-calories (kcal/kg)	−0.065	0.508
**Body weight gain status**		
## Change of daily body weight (gram)	0.093	0.306
### Variation of change of daily body weight (gram)	0.109	0.230

* GA, gestational age; §BBW, birth body weight, ‡ RDS, respiratory distress syndrome; ¶ PDA, patent ductus arteriosus; Ø NEC, necrotizing enterocolitis; Ð BPD, bronchopulmonary dysplasia; ð ROP, retinopathy of prematurity; † PMA, postmenstrual age; * Mean daily intake-calories within 3 days before incubator weaning; ** Mean difference of intake-calories within 3 days before incubator weaning; *** Variation of mean difference of intake-calories within 3 days before incubator weaning; ## Mean difference of body weight within 3 days before incubator weaning; ### Variation of mean difference of body weight within 3 days before incubator weaning.

## Data Availability

The data presented in this study are available on request from the corresponding author.
